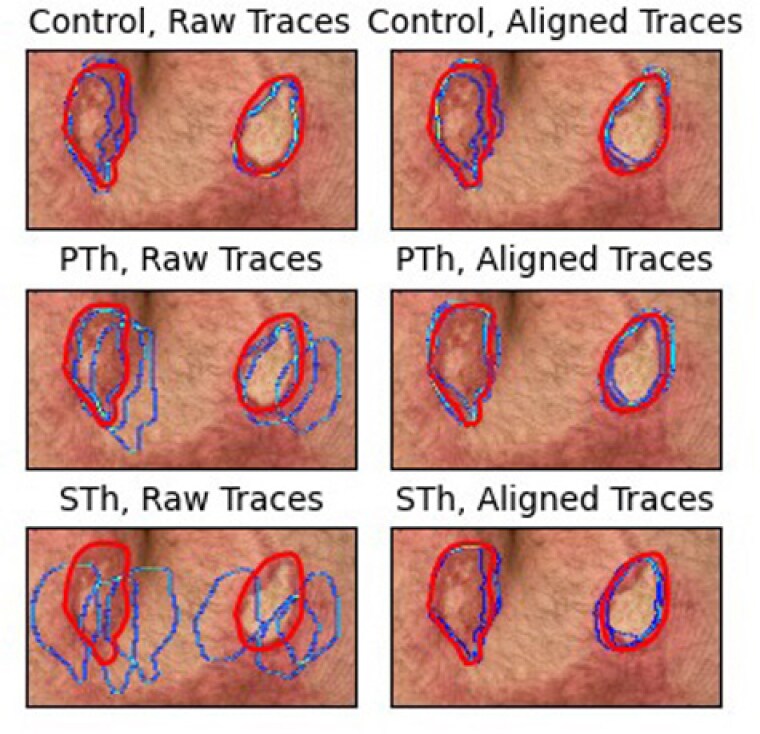# 814 Evaluation of Mixed Reality for Burn Margin Visualization and Surgical Planning

**DOI:** 10.1093/jbcr/iraf019.345

**Published:** 2025-04-01

**Authors:** Christopher Fedor, Griffin Hurt, Edward Andrews, Jacob Biehl, Francesco Egro

**Affiliations:** University of Pittsburgh School of Medicine; University of Pittsburgh; University of Pittsburgh Medical Center; University of Pittsburgh; University of Pittsburgh Medical Center

## Abstract

**Introduction:**

Mixed reality (MR) allows virtual content to be merged with the physical world, enabling novel visualization affordances not available with traditional displays. These affordances are particularly beneficial in the medical domain, as surgeons can view imaging and other relevant patient data in a 3D spatial context. In burn surgery, excision and grafting are mainstay treatments for deep partial and full-thickness burns. However, identifying regions to be excised is nontrivial. Recent work has developed imaging techniques that assist surgeons in determining burn margins. In this study, we demonstrate a new application of MR for burn surgery by building and evaluating a system that overlays deep burn margins onto a simulated anatomical surface for surgeons to trace. This represents a first step towards creating an MR system that can help physicians interpret burn surface area and depth for surgical planning.

**Methods:**

3 burn surgeons were asked to trace an MR-overlayed, pre-defined margin onto a printed image of a burn using two modern MR headsets: one see-through (STh) device and one pass-through (PTh) device. A control group was included, where surgeons drew the margins while referencing an image of the overlays on an external monitor. The headsets tracked the images using native libraries and 3 QR code fiducial markers placed in the top left, top right, and bottom left corners of the page. Accuracy was measured by intersection over union (IOU) between tracings and ground truth data. To control for inaccuracies in image tracking that can be resolved with known techniques, we also measured trace precision as IOU after aligning the curves with iterative closest point (ICP).

**Results:**

With the raw traces, average trace area IOU was highest for the control (0.718), then for the PTh headset (0.501), and lowest for the STh headset (0.187). When the traces were aligned with the ground truth using ICP, the pass-through headset had the highest IOU (0.797), followed by the see-through headset (0.770), with both outperforming the control condition (0.763) in trace precision.

**Conclusions:**

MR allows surgeons to more precisely mark a pre-defined burn margin than a monitor alone. Accurate image and object tracking is vital for the success of MR for surgical planning. We attribute the large differences in raw trace accuracy between the headsets to the inherently better tracking capabilities of the PTh headset software.

**Applicability of Research to Practice:**

Combining new diagnostic imaging approaches to automatically determine burn depth and the boundaries of burn excision, along with using MR as a visualization tool, may be able to decrease the size of burn excision and necessary skin grafts, ultimately improving patient outcomes.

**Funding for the Study:**

N/A